# Research on farmers’ households credit behavior and social capital acquisition

**DOI:** 10.3389/fpsyg.2022.961862

**Published:** 2022-11-22

**Authors:** Li Ping, Song Xiaosong, Li Jinzhao

**Affiliations:** ^1^Northeast Agricultural University, Harbin, China; ^2^Heilongjiang Academy of Social Sciences, Harbin, China

**Keywords:** business performance, farmer households’ credit behavior, social capital, agricultural eco-economy, empirical model (EM)

## Abstract

Farmer’s credit is significant to growing the farmer’s revenue and the progression of the rural economy. Several scholars have focused on the impact of social capital on the farmers’ credit availability, but the research deduction is still not combined. In addition, credit behavior is an important element that researchers pay attention to. However, the study mainly focuses on the effects of social capital on the credit behavior of farmer households and its large positive effect, a scientific approach for measuring farmer households’ social capital was presented. The strategy attempted to develop an empirical model of farmer households’ loan behavior based on business performance and the role of social capital. Through regression analysis of the influence of social capital on the channels of farmer households’ access to credit, it was determined that the error correlation coefficients of the credit by the formal channel and the informal channel were 0.153, 0.158, and 0.152, respectively, for the three models. The significance levels of the correlation coefficients were all greater than 1 percent, showing that the regression results were relatively accurate and passed the significance test. However, these results indicated that financial channels pay attention to farmers’ credit and social capital acquisition to reduce the risk of advancing. Moreover, informal money lenders such as friends and relatives focused on the social capital of the farmer. The study is helpful for policymakers to make the strategies for the farmer to get credit behavior from financial institutions. Moreover, this study is beneficial in highlighting some limitations and giving future directions to researchers.

## Introduction

The progress of the rural economy is not have happened without the provision of rural credits (Ali et al., 2014). A comparatively complete rural credit market can considerably increase the efficiency of farmers technically and enhance their income and utilization (Yanbo et al., 2018). However, today, the credit problem for farmers is still quite severe in China, especially the credit restraints from formal credit institutions. Therefore, (Kumar et al., 2013) uncovered that credit hurdles had a negative effect on farmers’ consumption, education, health, agricultural investment, and food. Moreover, the researchers have to pay attention to ways to help reduce farmers’ credit hurdles and enhance their credit availability. The effect of material and social capital on the availability of credit to farmers has also gained a noteworthy focus. Related research indicates that many reasons occur why farmers are the main focus of credit limitations, for instance, less effective mortgage and unflawed rural credit system as well as asymmetric comprehension between lenders and borrowers (Steijvers and Voordeckers, 2009). Moreover, they set the specific higher criteria for farmers to confirm that the borrower has the capacity to repay the loan and reduce the risk of lending (Liu et al., 2020). Therefore, better-off families have more access to get the loans to access because they have a strong ability to repay the loans (Malual and Mazur, 2017). Moreover, the farmers who belong to higher-income families treated a quality customers by banks and easily lent the money (Peng and Liu, 2016).

Further, Xu and Yuan (2017) reveals that better-off farmers have extra capital to capitalize on their social system as well as enhance their financial networks. However, the revenue of farmers is commonly low in China, material capital is not just only thing to use in an effective mortgage as well as social capital is important to use as additional to reduce the negative influence of material capital on credit availability to farmers (Liu et al., 2020).

**FIGURE 1:**
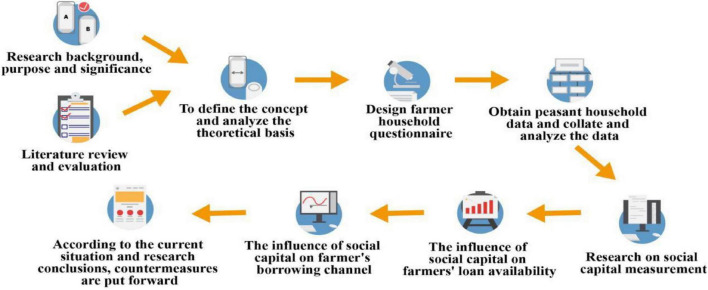
Technology route map.

In the 1990s, China’s urban, and rural binary contradiction was extremely obvious. Based on the situation, the policies to solve the “three rural issues” was put forward by China. On a large scale, there is a serious imbalance in China’s urban and rural economic development. And this kind of imbalance also indirectly restricts the increase of the social economy. Therefore, for the development of the rural economy in China, how to better balance urban and rural development and gradually narrow the gap is a major problem that needs to be solved as soon as possible (da Silva et al., n.d.). In recent years, with the deepening of poverty alleviation work, government departments and social institutions also have provided financial support for rural development. As shown in Figure 1.

China is a largely agricultural country, however, China’s production related to agricultural is small scale with low productivity and efficiency with rising the production budget. Further, agricultural production also confronts the natural and market risks. Consequently, farmers’ agricultural revenue is less and unbalanced. In such a way, financial organizations are commonly reluctant to lend to farmers. The agricultural capital investment cycle is long, the single business limit is low, and the natural risk is high. So, the security and profitability of credit funds are considered, but farmer households’ credit is not concerned. Farmer households’ credit has been intricately linked with poverty alleviation and economic development in China’s rural areas, so farmer households’ credit must be attached of immense importance, and farmer households’ credit behavior and social capital acquisition must be paid attention to Júnior et al. (2020). Moreover, the farmers under social pressure are more likely to obey norms. Exclusive China’s rural culture suggests specific China’s problems.

## Literature review

The research on social capital originates from the research on “capital” in social science and develops along the path of material capital, human capital, and social capital from the perspective of evolution (Chen et al., 2021). Material capital is external to the actor with a specific material form, including various forms of wealth. Initially, people’s understanding of capital is limited to material capital. Human capital was introduced into economic analysis in the 1960s. They believed that educated and trained health workers in the society determined the utilization rate of classical factors of production, thus putting forward the concept of human capital, which was internalized in actors, such as intelligence, appearance, technology, experience, and other personal abilities (Demari et al., 2020). However, sociologists believed that not only the material capital and human capital could bring convenience for people, but all kinds of contact and interaction between people also could have the same effect. It brought convenience to people’s actions, which was conducive to the realization of the goal. Social capital was also productive, which made the concept of social capital be added to the collection of concepts of capital (Fani et al., 2021). The study exposed that education, farming understanding, landholding, family size, monthly revenue, and ownership of land are important factors for farmers to access credit. The socio-economic element plays a significant role in accessing agricultural credit. Therefore, there is a need to credit policies that discourse the farmer’s problems related to risk (e Saqib et al., 2016).

As for China’s social capital and private credit research, some scholars believed that the generalized folk credit was the floorboard of all kinds of folk financial, namely all folk financial activities that did not come from the official formal financial institutions. In the narrow sense, private credit mainly refers to the credit between individuals and includes the credit from individuals to collective enterprises and other mutual fund organizations. It was found that most of the middle- and low-income farmer households were excluded from the formal financial market because they could not provide effective collateral to meet the requirements of rural formal financial institutions for credit. And a considerable number of them had to turn to private credit or even credit sharks. Therefore, in the daily life of farmer households, social capital was a valuable resource for farmer households. Its foundation and carrier are social relations and social networks, which play a key role in the process of farmer households getting credit. According to Akram and Routray (2013) the heterogeneity of associations, participation in local institutions, and level of generalized and institutional trust were recognized as the main social capital dimension affecting credit access.

Some researchers analyzed the phenomenon that villagers made up the deficit and tide over difficulties through social relations. It was found that social relations had a significant impact on the farmer households’ economic behaviors, especially the circle structure based on blood relations and centered on smallholders’ families, and the friendship credit thus extended accounted for a considerable proportion of credit in rural areas (Brito et al., 2015).

According to Saqib et al. (2018) experience, education, risk assessment, revenue, access to credit foundations and socio-economic factors have found a positive association in adopting agricultural lending. In the research on social capital and credit, all researchers agreed that social capital played a key role in both private credit and formal credit (Tong et al., 2021). However, most of this research focused on rural economics. The relationship between social capital and credit acquisition was investigated mainly through quantitative methods, which confirmed that different social capital had a positive role in farmer households’ formal and informal access to credit. However, this research lacks an in-depth analysis of the function mechanism of social capital in credit, especially lacking attention to the social relationship between borrowers and the interaction between borrowers (Andriani et al., 2022).

This study aims to investigate the impact of the social network, social trust, and participation on farmers’ credit behavior. The study has the originality to examine the social capital acquisition with farmers’ credit behavior in China there is a found significant gap in this area.

## Methodology

The study adopted a quantitative methodology to examine the farmer’s credit behaviors and social capital acquisition. The study used the positivism philosophy with the deductive reasoning approach. Moreover, China has not perfect financial system for the construction of rural. There are few credit institutions and unbalanced distribution systems in rural areas of China. There are more traditional and single financial services that cannot be effective according to farmers’ needs and are harshly limited by credit.

### Data collection and sampling

In this study, the target population is China, and the farmers were used as a sample. The cross-sectional data has been used in this study to test the farmer households’ credit behavior and social capital. In the research, we selected certain farmer households from seven towns of a county as sample data. And farmer household samples were selected by random sampling. A questionnaire survey on the credit situation by entering households and interviewing village cadres was conducted. After obtaining the data through one-to-one access, they are sorted out. The relevant data for the research was finally obtained after input. 550 questionnaires were collected in the survey. And 515 valid questionnaires were determined excluding invalid ones (lack of personal information such as age and gender), with an effective rate of 93.6%.

### Study variables

The study uses explanatory research to explain and examine the different variables’ effects on each other and get the conclusion from previously existing theories. In the research, social capital is an independent variable that is divided into the social network, social trust, and social participation. The dependent variable of the research is farmer households’ credit behavior, which is divided into two levels of the research. One is whether farmer households can get credit, namely, the availability of credit.

### Statistical techniques

The study used the SPASS software for the analysis of data. SPASS software is an automated formula prover for first logic with equality. Therefore, the input of prover is a first-order formula in our syntax. The study runs the different analysis to test the variables such as common factor analysis, KMO test and Bartley sphericity test, Variance of common factors, Rotation component matrix, Rotation component matrix, component score coefficient matrix, Descriptive statistical and empirical analysis and regression analysis.

## Results

### Measurement analysis of farmer households’ social capital

The specific measurement indexes of each dimension are different. When measuring social networks, the number of people who are in regular contact, the degree of contact with relatives and friends, and contact with government and bank staff are taken as indexes. When measuring social trust, the degree of trust in relatives, government staff and bank staff are taken as indexes. When measuring social participation, the frequency of participating in village collective activities, activities organized spontaneously by villagers, and weddings and funerals are taken as indexes (Ekeke et al., 2021). Then the index of social capital and its dimensions are calculated by the factor analysis method. The specific situation is shown in Table 1 below.

**TABLE 1:** Index system of farmer households’ social capital.

Dimension	Questions	Assignment
Social network	The number of people who are in regular contact (X_1_)	1 = Almost none; 2 = Less; 3 = Average. 4 = More; 5 = Many
	The degree of contact with relatives and friends (X_2_)	1 = Almost never; 2 = Occasionally; 3 = Average; 4 = More frequent; 5 = Always
	The degree of contact with government staff (X_3_)	1 = Almost never; 2 = Occasionally; 3 = Average; 4 = More frequent; 5 = Always
	The degree of contact with bank staff (X_4_)	1 = Almost never; 2 = Occasionally; 3 = Average; 4 = More frequent; 5 = Always
Social trust	The degree of trust in relatives and friends (X_5_)	1 = Very distrustful; 2 = Distrustful; 3 = Average. 4 = Trustful; 5 = Very trustful
	The degree of trust in government staff (X_6_)	1 = Very distrustful; 2 = Distrustful; 3 = Average. 4 = Trustful; 5 = Very trustful
	The degree of trust in bank staff (X_7_)	1 = Very distrustful; 2 = Distrustful; 3 = Average. 4 = Trustful; 5 = Very trustful
Social participation	The frequency of participating in village collective activities (X_8_:)	1 = Almost never; 2 = Occasionally; 3 = Average; 4 = More frequent; 5 = Always
	The frequency of participating in activities organized spontaneously by villagers (X_9_)	1 = Almost never; 2 = Occasionally; 3 = Average; 4 = More frequent; 5 = Always
	The frequency of participating in weddings and funerals (X_10_)	1 = Almost never; 2 = Occasionally; 3 = Average; 4 = More frequent; 5 = Always

### Model construction

The factor analysis method generally applies to many variables or multicollinearity problems. Its specific principle is to use a few common factors to reflect the connection between multiple variables. In the investigation of the effect of social capital factors on farmer households’ credit behavior (Kaihua, 2020; Latifa et al., 2020), the independent variable was the variable of social capital, and the ten variables were selected to explain the social capital. The variable number was more, and the collinearity problem was likely to exist between these variables. So, the factor analysis method was applied to measure social capital research in the research (Lei and Zeng, 2020; Li et al., 2021). There are three main steps of the factor analysis method, which are as follows. First, select variables and assess the correlation between variables to analyze whether they were suitable for using this method. Second, determine the common factor. The common factor and its name are determined by rotating the component matrix, which is a key step in factor analysis. Third, calculate the score. According to the value of the original variable and factor component coefficient matrix, the value of each common factor is calculated. The specific calculation method is as follows.


(1) $$\begin{array}{l} F_{1}=a_{11}X_{1}+a_{12}X_{2}+\ldots \ldots a_{1(n-1)}X_{\left(n-1\right)}+a_{1n}X_{n}\\ F_{2}=a_{21}X_{1}+a_{22}X_{2}+\ldots \ldots a_{2(n-1)}X_{\left(n-1\right)}+a_{2n}X_{n}\\ \ldots \ldots \\ \ldots \ldots \\ F_{m}=a_{m1}X_{1}+a_{m2}X_{2}+\ldots \ldots a_{m(n-1)}X_{\left(n-1\right)}+a_{mn}X_{n} \end{array}$$

The formula *F* represents the common factor extracted after rotation. *a* Represents the score coefficient of the initial variable component. And *X* represents the original variable. Finally, the social capital index is calculated according to the score of each common factor and its weight. The specific calculation formula is shown in Formula 2.


(2) $$\begin{array}{l} \text{social capital index}\\ \text{=}\frac{\sum_{i=1}^{3}\text{Each common factor}\,*\,\text{respective variance contribution rate}}{\text{total variance contribution}} \end{array}$$

### Factor analysis of social capital

Before the factor analysis, relevant tests should be conducted on the ten initial variables of the selected social capital, namely the KMO test and Bartley sphericity test. There is a certain classification standard here. The KMO test value is between 0 and 1. And the correlation between variables from 0 to 1 gradually increases, which makes it more suitable to use factor analysis to process data. To be specific, 0.5 is an intermediate value of the KMO test. If the test value is below 0.5, it is not suitable for this method. If the test value is between 0.5 and 0.6, it is far-fetched and only marginally suitable, and the effect is not good. If the test value is between 0.7 and 0.8, it is very suitable for this method and the result is relatively accurate. If the value is greater than 0.9, it is very suitable (Li et al., 2020; Liu et al., 2021). Before the test, to ensure the accuracy of the analysis results, the experimental data was processed and standardized. Then SPASS software was used for the test. The test results of the research are shown in Table 2 below:

**TABLE 2:** KMO and Bartley sphericity tests.

KMO		0.806
Bartley sphericity test	The approximate Chi-square	3582.656
	Df	45
	Sig	0.00

As can be seen from the above table, the KMO test value between variables is 0.805, between 0.8 and 0.9, which has passed the correlation test and is suitable for factor analysis. In the Bartley sphericity test, the significance is 0.000, less than 0.05. The variable passes the significance test, so this method can be used to aggregate factors, to obtain common factors. The next step is to extract the variance of the common factor. According to the extraction results, the common degree of variables X_1_ to X_10_ is above 0.5 and the maximum value is 0.960. And the average value is about 0.8, which has a beneficial effect. In other words, the contribution rate of the common factor to the variance of variable X is maintained at about 80%. Overall, the common factor has a strong ability to interpret each variable, which is representative (Maimunah and Kristiawan, 2020; Patel et al., 2020). The variance results of common factors obtained in SPASS are shown in Table 3 below.

**TABLE 3:** Variance of common factors.

Variables	Initial value	Extraction
X_1_	1.00	0.740
X_2_	1.00	0.836
X_3_	1.00	0.839
X_4_	1.00	0.850
X_5_	1.00	0.559
X_6_	1.00	0.851
X_7_	1.00	0.846
X_8_	1.00	0.922
X_9_	1.00	0.956
X_10_	1.00	0.961

The common factors are then extracted. First, eigenvalues need to be judged. The eigenvalues of each factor should be over one. The eigenvalues of common factors extracted in the research are 3.227, 2.839 and 2.284 respectively, which are all more than one (Sabadin et al., 2020; Si, 2021). The variance contribution rate of social science articles to extracted common factors should be over 70% and the variance contribution rate of extracted common factors in the research is 83.505%. The specific conditions of common factor extraction are shown in Table 4 below.

**TABLE 4:** The interpretation of total variance.

Ingredients	Initialize eigenvalues			Extract the sum of squares and load			Rotate squares and load		
			
	Total	Variance %	Cumulative %	Total	Variance %	Cumulative %	Total	Variance %	Cumulative %
1	4.257	42.573	42.573	4.257	42.573	42.573	3.227	32.273	32.273
2	2.454	24.543	67.116	2.454	24.543	67.116	2.839	28.391	60.664
3	1.639	16.389	83.505	1.639	16.389	83.505	2.284	22.842	83.505
4	0.587	5.875	89.38						
5	0.334	3.338	92718						
6	0.210	2.089	94.810						
7	0.197	1.958	96.764						
8	0.166	1.648	98.412						
9	0.116	1.249	99.559						
10	0.045	0.443	100						

**FIGURE 2:**
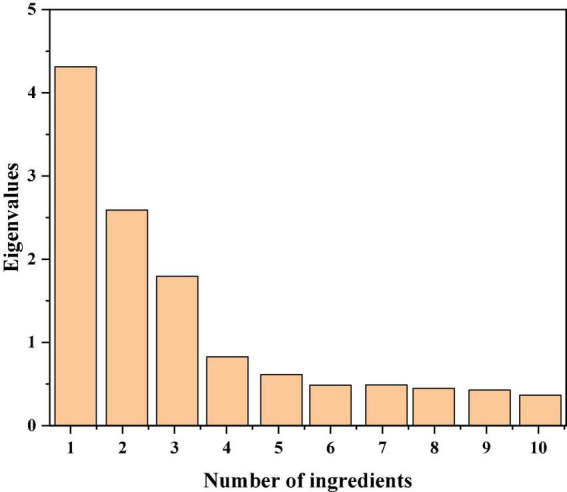
Scree plot.

As can be seen from the above table, three principal component factors are extracted for social capital variables. Considering that social capital contains many factors, which are not only limited to the factors designed in the research, but some factors are also not reflected in the research. It can be seen from Table 4 that the cumulative variance contribution rate of the total parties in the research is 83.505%, which is in line with expectations. It can be seen from Figure 2 in the research that the slope of the first three factors is large. Viewed directly from the vertical axis, the characteristic value is more than one. The curve slope of the latter factors gradually flattens out. Namely, three common factors are extracted in the research. The results got by SPASS data processing software are shown in Figure 2 below.

To reflect the common factors corresponding more clearly to each variable and explain the meaning of each common factor, then the component matrix is rotated. Variance maximization can be used to perform this operation in SPASS processing software to get the component rotation matrix. The results are shown in Table 5 below.

**TABLE 5:** Rotation component matrix.

Variables	Ingredients		
	
	F_1_	F_2_	F_3_
X_4_	0.902	0.058	0.178
X_2_	0.89	0.096	0.13
X_3_	0.865	0.116	0.107
X_1_	0.817	0.114	0.242
X_10_	0.117	0.972	0.037
X_9_	0.119	0.968	0.057
X_6_	0.083	0.954	0.062
X_7_	0.11	0.034	0.915
X_5_	0.215	0.064	0.892

As shown in Table 5, the load value of common factor F_1_ is the largest on X_1_, X_2_, X_3_ and X_4_. X_1_ represents the number of frequent contacts. X_2_ represents the degree of contact with friends and family. X_3_ represents the degree of contact with the government staff. X_4_ represents the degree of contact with the bank staff. So, it is named a social network. The load value of common factor F_2_ is the largest on X_8_, X_9_ and X_10_. X_8_ represents farmer households’ participation in village collective activities. X_9_ represents farmer households’ participation in villagers’ spontaneous organization activities. X_10_ represents farmer households’ participation in weddings and funerals. So, it is named social participation. On X_5_, X_6_ and X_7_, the load value of common factor F3 is the largest. X_5_ represents the trust of relatives and friends. X_6_ represents the trust of government personnel. X_7_ represents the trust of bank personnel. So, it is named social trust (Stute et al., 2020; Ume et al., 2020).

Next, the value of each common factor is calculated according to the value of the original variable and the component coefficient matrix of the factors. Finally, the social capital index is calculated according to the score and weight of each common factor. The scoring coefficient matrix is shown in Table 6 below.

**TABLE 6:** Component score coefficient matrix.

Variables	Ingredients		
	
	F_1_	F_2_	F_3_
X_1_	0.259	−0.02	−0.005
X_2_	0.311	−0.022	−0.087
X_3_	0.309	−0.03	−0.076
X_4_	0.304	−0.45	−0.05
X_5_	−0.05	−0.012	0.341
X_6_	−0.102	−0.017	0.448
X_7_	−0.061	−0.012	0.419
X_8_	−0.045	0.347	−0.004
X_9_	−0.032	0.35	−0.012
X_10_	−0.03	0.352	−0.022

According to the table above, the specific scores of the three common factors are as follows.


(3) $$F_{1}=0.258*X_{1}+0.311*X_{2}+\ldots \ldots +(-0.030)*X_{10}$$

(4) $$F_{2}=(-0.020)*X_{1}+(-0.022)*X_{2}+\ldots \ldots +0.352*X_{10}$$

(5) $$F_{3}=(-0.005)*X_{1}+(-0.087)*X_{2}+\ldots \ldots +(-0.022)*X_{10}$$

The final social capital index is calculated as shown in Formula 6.


(6) $$F=(32.273*F_{1}+28.391*F_{2}+22.842*F_{3})/83.505$$

Social network accounts for the largest proportion of social capital, accounting for 32.273%, which also indicates that social network is the most important for farmer households to establish their social capital. It is followed by social participation, accounting for 28.391. The higher the participation of farmer households, the more extensive human resources, and the higher the social capital. The final is social trust, accounting for 22.842%, which is the smallest proportion of social capital.

### Descriptive statistical

(1) Investigation of farmer households’ credit behavior.

In terms of age distribution, farmer households between 40 and 60 years old are the most enthusiastic about credit, probably because they are middle-aged and have rich life experience. In terms of the distribution of education levels, the credit behavior of farmer households with junior middle school to senior high school education levels is more, reflecting the difference in credit behavior of farmer households with different education levels in a county. As shown in Table 7.

**TABLE 7:** Farmer household household credit.

Classification type	Distribution options	Formal credit	Informal credit	Non-participation in credit
Age distribution	Less than 40 years old	2	12	27
	40–60 years old	16	54	240
	More than 60 years old	5	24	138
Education level distribution	Primary school or below	1	26	116
	Junior high school or senior high school	18	55	223
	Senior high school or above	4	9	66

(2) Analysis of the use of farmer households’ credit.

**FIGURE 3:**
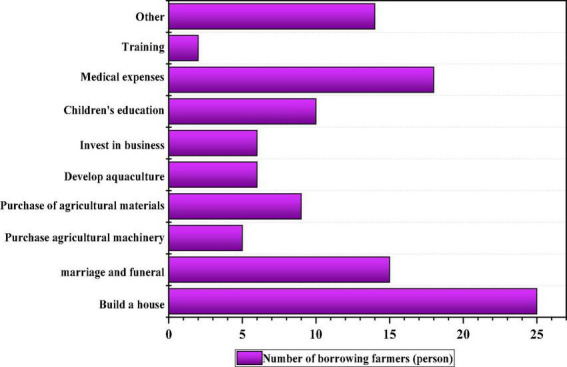
Use of farmer households’ credit.

The purposes of credit can be roughly divided into ten categories, including building a house, weddings and funerals, purchase of agricultural machinery, purchasing agricultural materials, developing aquaculture, investment and business, children’s education, medical expenses, and skills training, and others (Zheng et al., 2020). Among all the sample farmer households with credit behavior, building a house and medical expenses account for the largest proportion, which makes up the main motivation of farmer households’ credit. As shown in Figure 3.

(3) Analysis of farmer households’ credit channels.

As can be seen from Table 8, many farmer households prefer informal credit channels, accounting for 79.1%, while those who borrow credit formally only account for 18.2%. farmer households choose more informal credit channels to meet their financing needs because formal financial institutions such as banks set strict credit thresholds, while farmer households lack certain collateral and complicated bank credit procedures, which to some extent hinder farmer households from obtaining credit from formal financial institutions.

**TABLE 8:** Analysis of credit channels.

Classification	Frequency	Percentage
Formal credit	23	4.5
Informal credit	90	17.5
Non-participation in credit	405	78.6

(4) Analysis of the relationship between family members’ work in enterprises and farmer households’ credit behavior.

It can be obtained from the proportion of credit for enterprise workers and non-enterprise workers in the total sample credit that families the formal credit rate of enterprise workers is higher than the non-enterprise workers, reaching 26.7%. Perhaps enterprise workers have more relevant information and resources of the financial institution’s credit, to have more opportunities for formal institutions’ credit. However, in terms of informal credit channels, the proportion of non-enterprise workers’ credit is higher than family members working in enterprises, reaching 17.7%. See Table 9 for details.

**TABLE 9:** Analysis of the relationship between enterprise workers and credit behavior.

	Formal credit		Informal credit		Non-participation in credit	
			
Characteristic	Frequency	Percentage	Frequency	Percentage	Frequency	Percentage
Enterprise workers	8	26.7	4	13.3	19	63.3
Non-enterprise workers	15	3.1	86	17.7	386	79.6

### Empirical analysis

#### Descriptive statistics of variables

The dependent variable of the research is farmer households’ credit behavior, which is divided into two levels of the research. One is whether farmer households can get credit, namely, the availability of credit. The other is the channels for farmer households to obtain credit. Specifically, the availability of credit refers to whether farmer households can obtain credit in real life and the research of credit channels refers to whether farmer households can obtain credit through formal or informal channels (Yehuala, 2008). The details are shown in Table 10.

**TABLE 10:** Variable description and descriptive statistics.

	Variable name	Assignment	Average value	Standard rate
The characteristics of the household head	Age (X_1_)	Actual value	52.70	13.34
	Gender (X_2_)	0 = Male; 1 = Female	0.39	0.49
	Education Level (X_3_)	1 = without education. 2 = Primary school. 3 = Junior high school. 4 = Senior high school. 5 = Junior college. 6 = University or above	2.39	1.25
	Health (X_4_)	1 = Serious illness or loss of Labor force. 2 = Suffering from disease. 3 = General. 4 = Good. 5 = Healthy	3.84	1.21
Family characteristics	Total household size (X_5_)	Actual value	4.92	1.68
	Household labor force (X_6_)	Actual value	2.47	1.24
	Total household income (X_7_)	Actual value (*Ten thousand Yuan*)	9.11	15.79
Credit characteristics	Policy Understanding (X_8_)	1 = Completely not understand; 2 = Understand a little; 3 = Generally understand; 4 = Understand. 5 = Completely understand	2.48	1.35
	Credit facility (X_9_)	0 = Inconvenient. 1 = Convenient	0.75	0.44
Social capital	Social network (X_10_)	A value calculated by factor analysis	3.77	1.07
	Social trust (X_11_)	A value calculated by factor analysis	3.74	1.01
	Social participation (X_12_)	A value calculated by factor analysis	3.64	1.21
	Social capital index (X_13_)	A value calculated by factor analysis	3.72	1.10
The dependent variable	Credit availability (Y_1_)	0 = Unavailable; 1 = Available	0.70	0.46
	Formal financial credit institutions (Y_2_)	0 = No; 1 = Yes	0.47	0.50
	Informal credit channel (Y_3_)	0 = No; 1 = Yes	0.33	0.47

#### Model construction

In the research, the effect of social capital on farmer households’ credit is investigated. And the farmer households’ credit is divided into credit availability and credit channel, respectively. The research of the dependent variable is always binary classification variables, so there are only two values, namely yes or no. So, in the research, the binary logistic regression model is selected in the empirical part of the regression analysis. Logistic regression analysis is a common regression analysis method, which is often used in medical research, economic forecasting, and other fields. In the results of binary logistic regression, we can intuitively see the significant results and influence coefficients of each variable on the dependent variable. The model expression constructed in the research is as follows.


(7) $$Logistic=Ln\left(\frac{p_{i}}{1-p_{i}}\right)$$

(8) $$Logistic=\beta_{0}+\beta_{1}K+\beta_{2}S+\varepsilon$$

In the formula, *K* is the control variable, specifically referring to the characteristics of farmer households, family characteristics and credit characteristics.*S* is the core explanatory variable, namely farmer households’ social capital? ε is the random interference term. In the regression analysis to explore the influence of social capital on whether farmer households get credit or not, the dependent variable *y* = 0 means that farmer households do not get credit and *y* = 1 means that farmer households get credit. In the regression analysis of the influence of social capital on farmer households’ access to credit channels, the dependent variable *y* = 0 is set as no and *y* = 1 is set as yes for the influence of formal financial channels. For the influence of informal financial channel credit, the dependent variable*y* = 0 is also set as no and *y* = 1 is set as yes.

#### Regression analysis

(1) Binary Logistic regression analysis.

Before the regression analysis, the variance inflation factor (VIF) was applied to the collinearity test using Stada12.0 data processing software. And the results showed that the maximum VIF was 1.52, which was less than 10. Therefore, there was no such problem and model regression could be continued. Then, binary Logistic regression analysis was conducted, and the specific results were shown in Table 11 below.

**TABLE 11:** Regression analysis of social capital on farmer households obtain credit.

Variable	Explained variable: Availability of credit for farmer households		
	
	Model (1)	Model (2)	Model (3)
The householder ages	−0.043**	−0.068**	−0.057*
	(0.025)	(0.033)	(0.027)
Gender (Female)	0.527	1.040	0.510
	(0.669)	(0.730)	(0.579)
Education Level	0.728**	0.675**	0.654**
	(0.270)	(0.296)	(0.263)
Health	0.757**	0.881**	0.927**
	(0.295)	(0.333)	(0.307)
Total household size	−0.312	−0.334	−0.085
	(0.238)	(0.247)	(0.223)
Labor force	0.404	0.480	0.239
	(0.314)	(0.342)	(0.305)
Household income	0.215***	0.207*	0.188**
	(0.204)	(0.089)	(0.073)
Understanding of loan policies		0.840***	0.782***
		(0.279)	(0.250)
Credit facility		0.026	0.013
		(1.006)	(0.078)
Social network	4.369***	4.611***	
	(0.639)	(0.741)	
Social trust	2.857***	3.115***	
	(0.484)	(0.562)	
Social participation	1.974***	2.119***	
	(0.410)	(0.485)	
Social capital			8.875***
			(1.299)
Constant	−7.331***	−7.331***	−5.769***
	(2.576)	(2.576)	(2.185)

In the table, passing the significance test of 10% is represented by *. Passing the significance test of 5% is represented by **. Passing the significance test of 1% is represented by ***. The data in parentheses is the standard deviation.

(2) Analysis of the empirical results.

Firstly, model (1) controls the household head characteristics and family characteristics of the farmer’s household. And then the influence of different dimensions of social capital on the availability of credit for farmer households is analyzed. Between the two controlled characteristics, the household head’s education level, health level and household income have a positive effect on the availability of credit to the farmer household, which passes the significance level test of 5%. While the household head’s age has a negative effect, which also passed the significance level test of 5%. However, the gender of the household head, the number of family members and the number of the labor force have little relation to whether the farmer’s household can obtain credit, which fails to pass the test. This shows that the older the farmer’s household head is, the lower his ability to obtain wealth through labor in the future, and the weaker his ability to repay the credit, so the possibility of obtaining credit will be reduced. The higher the level of education, the higher the ability to actively learn new knowledge and the higher the level of understanding, the deeper the understanding of credit policies, procedures and procedures, the more information they master, and the more flexible their thinking, and the stronger the demand for production and investment, so the more inclined they are to borrow credit. The better the health of the household head, the stronger the repayment ability. While the farmer households’ labor ability with the long-term illness will be weak or even lost. They are unable to guarantee their livelihood and the repayment ability will be worse, so it is not easy to obtain credit. With every increase of one unit in household income, the possibility of farmer households getting credit will increase by 0.215 units, showing a positive correlation. The higher the income, the stronger the demand for production and operation, and the more inclined they are to borrow credit. The insignificant influence of the other two factors on family characteristics may be due to the absolute amount investigated in the research, rather than the relative amount. For the amount investigated, it may be more appropriate to investigate the proportion of the labor force in families. Among the core explanatory variables, the three dimensions of social capital all pass the significance level test of 1%. Social network, social trust and social participation all have a positive impact on the possibility of the farmer’s household obtaining credit, which is consistent with the expectation. The wider the social network of the farmer household is, the more people they contact regularly. The closer they contact relatives and friends, government officials and bankers, the more information they will get. The smaller the risk of information asymmetry will be, the higher the possibility of the farmer’s household obtaining credit. In terms of social trust, the more trust farmer household households have in relatives and friends, government staff and bank staff, the lower the transaction cost will be, and the more they can obtain credit. The higher the social participation of farmer households, the lower the risk of information asymmetry and the higher the probability of obtaining credit.

Secondly, based on the model (1), considering the impact of characteristics of farmer households’ credit themselves on farmer households’ credit availability and on the result of the model, to avoid deviation, the characteristics of farmer households’ credit are added in the model (2), namely the farmer households’ understanding of the credit policy and the convenient degree of credit. The results show that the significant influence of each dimension of social capital on farmer households’ credit availability has not changed and it is still significant, except that the coefficient and standard deviation have changed. As for credit characteristics, only the understanding of credit policy passes the test of significance of 1%, having a significantly positive impact on farmer households’ obtaining credit. It shows that the more farmer households know the related policy, and financial institutions, the communication between the relatives and friends and related organization degree, the better communication degree, which is conducive to obtaining credit. However, the convenience of credit does not pass the significance test, which may be since the rural areas in the research are scattered intensively and there is no great geographical difference, so the convenience of credit is similar.

Thirdly, model (3) replaces the core explanatory variable with the total social capital index based on Model (2). The influence of the total social capital index on the credit availability of farmer households is separately investigated and overall analysis is made. The impact of the total social capital index on farmer households’ access to credit has a significant impact of 1%, which is still significant overall. This indicates that the higher the social capital index is, the stronger the social resources, relationship network and ability to fulfill contracts are, and the more likely farmer households will obtain credit.

#### Regression analysis

According to the dual finance theory and considering that social capital may have different influences on farmer households’ channels of obtaining credit, farmer households’ choice of different credit channels will have different influences on the development of rural finance in China. Therefore, this chapter of empirical research is to investigate the influence of social capital and its various dimensions on farmer households’ credit channels, respectively. The model regression results of social capital and its influences on farmer households’ credit channels are shown in Table 12 below.

**TABLE 12:** Social capital on farmer households’ credit channels.

Variable	Formal credit channel			Informal credit channel		
		
	Model (1)	Model (2)	Model (3)	Model (1)	Model (2)	Model (3)
The householder ages	−0.011**	−0.023*	−0.023*	−0.019*	−0.005	−0.003
	(0.011)	(0.012)	(0.012)	(0.01)	(0.011)	(0.01)
Gender (Female)	−0.419	−0.263	−0.301	0.745**	0.507*	0.421
	(0.296)	(0.311)	(0.308)	(0.264)	(0.279)	(0.270)
Education Level	0.203***	0.249***	0.254***	0.262***	0.199***	0.204**
	(0.113)	(0.124	(0.123)	(0.095)	(0.103)	(0.122)
Health	0.275**	0.274**	0.296**	0.317***	0.380*	0.358***
	(0.131)	(0.137)	(0.135)	(0.122)	(0.127)	(0.122)
Total household size	−0.069	−0.003	−0.001	−0.120	−0.189**	−0.172*
	(0.084)	(0.107)	(0.003)	(0.086)	(0.092)	(0.088)
Labor force	0.107	0.082	0.080	0.104	0.089	0.088
	(0.137)	(0.143)	(0.142)	(0.118)	(0.122)	(0.120)
Household income	0.014***	0.018**	0.019**	0.012**	0.018***	0.018***
	(0.012)	(0.017)	(0.018)	(0.007)	(0.007)	(0.07)
Understanding of loan policies		0.349***	0.357***		−0.299***	−0.254**
		(0.114)	(0.114)		(0.107)	(0.103)
Credit facility		1.000**	1.297**		1.840***	1.263**
		(0.409)	(0.381)		(0.404)	(0.359)
Social network		1.315***		0.591***	0.891***	
		(0.192)		(0.149)	(0.169)	
Social trust		0.978***		0.264**	0.384**	
		(0.162)		(0.132)	(0.141)	
Social participation		0.842***		0.132	0.126	
		(0.164)		(0.124)	(0.112)	
Social capital			3.046***			1.125**
			(0.355)			(0.267)
Constant	−2.992***	−3.505***	−3.370	−3.928***	−4.634***	−4.228***
	(1.048)	(1120)	(1.115)	(0.940)	(1.057)	(1.009)

In the table, passing the significance test of 10% is represented by *. Passing the significance test of 5% is represented by **. Passing the significance test of 1% is represented by ***. The data in parentheses is the standard deviation.

#### Results analysis

Firstly, in the model’s (1) control variables, the household head’s age and family income have a greater impact on the formal credit channel. Gender and health status of the household head have a greater impact on the informal credit channel. The degree of schooling has the same effect. The number of families and laborers is still insignificant. The age of the head of the farmer’s household has a detrimental effect on the household’s access to the two credit channels. The greater the age of the head of the household, the more uncertain the future will be. Therefore, the risk is increased regardless of whether relatives and friends or financial institutions are unwilling to give money.

For farmer households to borrow credit from informal channels, the gender of the household head meets the significance test of 5 percent, however, a female household head has a negative influence on credit from formal financial institutions and fails the significance test. They are more likely to borrow from family and friends, which may be related to the greater conservatism of the female head of household. Moreover, compared to its influence on the other two channels, farmer households’ social capital has a stronger impact on their loans from formal financial institutions, particularly in terms of social trust and social involvement.

Secondly, after adding credit characteristics into the model (2), the significant impact of each dimension of social capital on farmer households’ access to the two credit channels does not change. At this point, the effects of the credit characteristics of farmer households on the formal and informal channels to obtain credit both pass the test of significance of 1%. But the effect of the understanding of credit policy on the credit obtained from the informal financial channel is negative significantly, it may be because the more farmer households understand the credit policies, the more familiar they are with the procedures of borrowing credit from formal financial institutions.

Thirdly, in model (3), the influence of the total social capital index on different credit channels of farmer households is investigated. It can be seen from Table 12 that, overall, the total social capital index of farmer households has a positive and significant impact on credit through different channels. But comparatively speaking, it has a greater impact on farmer households’ obtaining credit from formal financial institutions, which is different from the impact of various dimensions of social capital. Therefore, it is confirmed once again that the influence of each dimension should be specifically analyzed when analyzing the influence of social capital on farmer households’ credit channels. The empirical results are shown in Table 13 below.

**TABLE 13:** Results of the two-variable probit model.

Variable	Formal credit channel			Informal credit channel		
		
	Model (1)	Model (2)	Model (3)	Model (1)	Model (2)	Model (3)
Social network	0.761***	0.667***		0.304***	0.469***	
Social trust	0.542***	0.504***		0.105*	0.186*	
Social participation	0.467***	0.455***		0.071	0.026	
Social capital			1.625***			0.606**
Control variables	Control	Control	Control	Control	Control	Control
The correlation coefficient between the error terms	0.153***	0.158***	0.152***	0.153***	0.158***	0.152***
Waid test: chi^2^1	86.67	76.63	71.75	86.68	76.64	71.74

In the table, passing the significance test of 10% is represented by *. Passing the significance test of 5% is represented by **. Passing the significance test of 1% is represented by ***. The data in parentheses is the standard deviation.

As can be seen from the table above, the correlation coefficients of error terms between credit models (1), (2) and (3) of the two channels are 0.153, 0.158 and 0.152, respectively. The errors among the three models are exceedingly small and the correlation coefficients of the three models all pass the significance level of 1%. Therefore, it can be shown that simply using two binary Logistic models for regression analysis will indeed cause certain errors. To reflect the impact of social capital more accurately on farmer households’ credit channels, the Bprobit model is used to evaluate again. Compared with the regression results of the binary Logistic model, the degree of influence of social capital on the possibility of credit through the two channels is reduced, but it still passes the significance test. Therefore, the empirical regression results in the research are relatively accurate.

The stability of the empirical regression results is evaluated by the method of the replacement model. According to the distribution characteristics of variables in the research, the Probit model is used instead of the binary logistic model for the robustness test in this chapter and the control variables are no longer displayed. The test results are shown in Tables 14, 15 below.

**TABLE 14:** Robustness test of social capital on the availability of credit to farmer households.

Variable	Explained variable: Availability of credit for farmer households		
	
	Model (1)	Model (2)	Model (3)
Social network	2.387***	2.522***	
	(0.320)	(0.375)	
Social trust	1.557***	1.684***	
	(0.244)	(0.281)	
Social participation	1.113***	1.188***	
	(0.217)	(0.264)	
Social capital			4.905***
			(0.670)
Control variables	Control	Control	Control

In the table, passing the significance test of 10% is represented by *. Passing the significance test of 5% is represented by **. Passing the significance test of 1% is represented by ***. The data in parentheses is the standard deviation.

**TABLE 15:** Robustness test of social capital on farmer households’ availability of credit.

Variable	Formal credit channel			Informal credit channel		
		
	Model (1)	Model (2)	Model (3)	Model (1)	Model (2)	Model (3)
Social network	0.850***	0.761***		0.361***	0.518***	
	(0102)	(0.106)		(0.088)	(0.096)	
Social trust	0.612***	0.553***		0.161**	0.242**	
	(0.093)	(0.106)		(0.078)	(0.082)	
Social participation	0.424***	0.488***		0.085	0.101	
	(0.082)	(0.092)		(0.074)	(0.093)	
Social capital			1.758***			0.670**
			(0.192)			(0.155)
Control variables	Control	Control	Control	Control	Control	Control

In the table, passing the significance test of 10% is represented by *. Passing the significance test of 5% is represented by **. Passing the significance test of 1% is represented by ***. The data in parentheses is the standard deviation.

Whether investigating the influence of social capital and its various dimensions on the availability of farmer households’ credit or the channels of farmer households’ credit, it can be seen from the two tables above that the influence of the core explanatory variable on the explained variable after the replacement model is consistent with the significance results before the replacement, except for differences in coefficient and standard error. Therefore, the model estimation in the research can be regarded robust.

## Discussion

Characteristics, the household head’s education level, health level and household income have a positive effect on the availability of credit to the farmer household, while the household head’s age has a negative effect (Sertse et al., 2021).

This shows that the older the farmer’s household head is, the lower his ability to obtain wealth through labor in the future, and the weaker his ability to repay the credit. The higher the level of education, the higher the ability to actively learn new knowledge and the higher the level of understanding, the deeper the understanding of credit policies, procedures and procedures, the more information they master, and the more flexible their thinking, and the stronger the demand for production and investment, so the more inclined they are to borrow credit (Zhao et al., 2021).

The insignificant influence of the other two factors on family characteristics may be due to the absolute amount investigated in the research, rather than the relative amount. For the amount investigated, it may be more appropriate to investigate the proportion of the labor force in families (Wang et al., 2021).

The wider the social network of the farmer household is, the more people they contact regularly. The closer they contact relatives and friends, government officials and bankers, the more information they will get. The smaller the risk of information asymmetry will be, the higher the possibility of the farmer’s household obtaining credit. In terms of social trust, the more trust farmer household households have in relatives and friends, government staff and bank staff, the lower the transaction cost will be, and the more they can obtain credit. The higher the social participation of farmer households, the lower the risk of information asymmetry and the higher the probability of obtaining credit (Ouattara et al., 2022).

The results show that the significant influence of each dimension of social capital on farmer households’ credit availability has not changed and it is still significant, except that the coefficient and standard deviation have changed (Kehinde et al., 2021).

The influence of the total social capital index on the credit availability of farmer households is separately investigated and overall analysis is made. The impact of the total social capital index on farmer households’ access to credit has a significant impact of 1%, which is still significant overall. This indicates that the higher the social capital index is, the stronger the social resources, relationship network and ability to fulfill contracts are, and the more likely farmer households will obtain credit (He and Ahmed, 2022).

The household head’s age and family income have a greater impact on the formal credit channel. Gender and health status of the household head have a greater impact on the informal credit channel. The degree of schooling has the same effect. The number of families and laborers is still insignificant. The age of the head of the farmer’s household has a detrimental effect on the household’s access to the two credit channels. The greater the age of the head of the household, the more uncertain the future will be. Therefore, the risk increases regardless of whether relatives, friends, or financial institutions are unwilling to give money (Ruml and Parlasca, 2022).

The significant impact of each dimension of social capital on farmer households’ access to the two credit channels does not change. At this point, the effects of the credit characteristics of farmer households on the formal and informal channels to obtain credit (Si et al., 2021).

the influence of the total social capital index on different credit channels of farmer households is investigated. Farmer households’ total social capital index has a positive and significant impact on credit through different channels. Therefore, it is confirmed once again that the influence of each dimension should be specifically analyzed when analyzing the influence of social capital on farmer households’ credit channels (Pham and Mukhopadhaya, 2022).

## Conclusion and recommendations

Under the supervision of the theory of farmer households’ economic behavior, the theory of farmer households’ credit, and the theory of social capital, the relevant domestic and international literature was combed through and summarized. Combining qualitative and quantitative analysis, a study on the influence of social capital on the credit behavior of farmer household households in a county was conducted by analyzing the summer survey data of farmer household households’ credit. The most important conclusions are as follows.

Firstly, farmer households’ social capital has a significant and positive impact on the availability of credit.

Second, diverse types of social capital have distinct effects on the credit channels of farmer households. The results of the analysis of the two-variable probit model indicate that the error correlation coefficients of the credit of formal institutions and informal channels between the three models are 0.153, 0.158, and 0.152, respectively, and the significance levels of the correlation coefficients are all greater than 1%, indicating that the regression results have passed the significance test and are reasonably accurate.

Thirdly, social capital has a stronger impact on farmer households’ access to formal financial institutions than informal loan channels. Social capital is intangible, meaning that it is not a physical substance. However, it can serve as collateral to increase the likelihood of formal banking institutions extending credit to farming households.

This study’s findings revealed a significant relationship between social capital and farmers’ credit behavior. This study suggested and encouraged the government and other agencies. Therefore, the current social capital should be implemented in the design of programs related to microfinance and utilized in rural areas’ development activities. The study is also helpful for the policymakers to invest in the creation of social capital activities and directly in the environmental supporting programs and their creation. Moreover, there is necessary for the policymakers to address the credit policies and issues of farmers that are facing the risk. Existing credit policies should be amended to shelter the interest of the farmers who lack security. The study suggested some future research that should be conducted in the future by other scholars. The study is limited to China but could be in other countries or regions. The study tested the social capital with credit behaviors of farmers that could be tested with other variables in the future.

## Data availability statement

The original contributions presented in this study are included in the article/supplementary material, further inquiries can be directed to the corresponding author.

## Author contributions

LP wrote introduction section. SX wrote the review. LJ did analysis. All authors contributed to the article and approved the submitted version.
